# Multi-Response Optimization of Face Milling Performance Considering Tool Path Strategies in Machining of Al-2024

**DOI:** 10.3390/ma12071013

**Published:** 2019-03-27

**Authors:** Raneen Abd Ali, Mozammel Mia, Aqib Mashood Khan, Wenliang Chen, Munish Kumar Gupta, Catalin Iulian Pruncu

**Affiliations:** 1 College of Mechanical and Electrical Engineering, Nanjing University of Aeronautics and Astronautics, Nanjing 210016, China; engraneen@nuaa.edu.cn (R.A.A.); dr.aqib@nuaa.edu.cn (A.M.K.); cwlme@nuaa.edu.cn (W.C.); 2 Mechanical and Production Engineering, Ahsanullah University of Science and Technology, Dhaka 1208, Bangladesh; mozammelmiaipe@gmail.com; 3 University Center for Research & Development, Chandigarh University, Gharuan 140413, Punjab, India; munishguptae7876@cumail.in; 4 Mechanical Engineering, Imperial College London, Exhibition Rd., London SW7 2AZ, UK; 5 Mechanical Engineering, School of Engineering, University of Birmingham, Birmingham B15 2TT, UK

**Keywords:** face milling, surface roughness, grey relation analysis, tool path strategy, multi-objective optimization

## Abstract

It is hypothesized that the orientation of tool maneuvering in the milling process defines the quality of machining. In that respect, here, the influence of different path strategies of the tool in face milling is investigated, and subsequently, the best strategy is identified following systematic optimization. The surface roughness, material removal rate and cutting time are considered as key responses, whereas the cutting speed, feed rate and depth of cut were considered as inputs (quantitative factors) beside the tool path strategy (qualitative factor) for the material Al 2024 with a torus end mill. The experimental plan, i.e., 27 runs were determined by using the Taguchi design approach. In addition, the analysis of variance is conducted to statistically identify the effects of parameters. The optimal values of process parameters have been evaluated based on Taguchi-grey relational analysis, and the reliability of this analysis has been verified with the confirmation test. It was found that the tool path strategy has a significant influence on the end outcomes of face milling. As such, the surface topography respective to different cutter path strategies and the optimal cutting strategy is discussed in detail.

## 1. Introduction

Due to the importance of the finishing stage in the manufacturing processes, the face milling process is the solution that can be used to achieve good surface quality and high accuracy in a short period of time. To achieve the high quality for the desired parts, studying the tool path strategies is inevitable too. The most common cutter path strategies in the milling process are zig, zig-zag, and contour, which can be created with the help of a computer-aided manufacturing system (CAM). Furthermore, the tool path generation is the prime issue in the different stages of NC machining that determines not only the quality of the desired shapes but also the performance of the manufacturing process [1,2]. Consequently, the optimization of tool path would contribute to improving the sufficiency of the milling process [3].

The application and selection of tool path types and directions are crucial issues in the milling of the die and aerospace industries. Moreover, the intact selection may lead to reduce the machining time and enhance the surface quality of the milling parts, hence leading to higher productivity and lower product costs. Toh [1] addressed a comprehensive review of the three most common cutting path strategies, i.e., offset, zig, zig-zag. The analytical study, effect of tool angle at the entrance and exit, and the inclined milling effects were evaluated with respect to the three previous tool path strategies. The better surface quality and optimum tool life were found with the inclination angle of 15°. Monreal and Rodriguez [2] studied the effect of tool path strategy on the cyclic time in the high-speed milling process. Based on experimental observation, the mechanical model is constructed to evaluate the cyclic time for a raster path strategy. Rangarajan and Dornfeld [3] showed that the features of part orientation and tool path in face milling operation with 10%–20% saving in cyclic time by using the feed rate profile. Lazoglu et al. [4] introduced a new approach to generate the optimum tool path strategy for free-form surfaces. The optimization process was based on the determination of the tool path with minimum machining force which should not exceed the maximum limit. Kim et al. [5] proposed an algorithm to optimize the contour tool path while considering the cutting force and vibration. The optimization was performed for two-dimensional parts with a flat end mill tool. The material removal rate and cutting forces were kept constant to prevent the vibration in the milling area. Ramos et al. [6] studied the influence of three tool path strategies called radial, raster and 3D offset on the surface roughness, texture and dimensional deviations of the free-form surface. They revealed that the milling time was not significantly different for the used strategies. However, the last strategy showed the best surface finish, uniform texture and dimensional performance, which confirmed the dependency of this strategy in free form surface machining. Quinsat and Sabourin [7] developed a methodology to select the optimal tool path strategy with a guarantee of high-level surface quality. This methodology based on the directional beams, which represented the feed directions to ensure the maximum performance for the strategy chosen.

Recently, due to increasing demand for manufacturing the complex parts with a large scale, the robotic milling system is used to perform this function. In this trend, many works have been achieved to improve the surface roughness and geometrical deviations, taking into consideration the optimum selection of cutter path strategies which confirmed the significance of the tool path not limited to the CNC milling process but also to the robotic system. Unnikrishna et al. [8] studied the tool path strategies of Al6005A alloy in the milling process of the 6-axis robotic system. The optimization has been performed using the Taguchi-Grey relational method. Tunc and Stoddart [9] addressed the tool path patterns in robotic milling. In this study, the zig tool path in two different feed directions and contour tool path are considered.

Conventionally, the optimization of process parameters is based on the trial-and-error method—it needs more time and cost. Hence, the optimization technique, which is able to predict the quality and quantity of machining characteristics, is required. Balajia et al. [10] optimized drilling parameters of Ti-6Al-4V on surface roughness, flank wear and drill vibration using response surface methodology. Similarly, the study performed by Mia et al. [11] used the Taguchi based GRA methodology to obtain the optimum turning parameters of AISI 4140 that gave the best surface roughness for the machined parts.

Taguchi based grey relation analysis has been adopted to predict surface roughness due to the versatility of this technique in different industrial processes. Asiltürk and Akkuş [12] have carried out the CNC turning experiment using the Taguchi method in order to minimize the surface roughness. The Taguchi method for finding out the optimal value of surface roughness under an optimum cutting condition in turning SCM 440 alloy steel was applied by Thamizhmanii and Sulaiman [13]. Recently, multi-objective optimization was used to enhance the surface roughness and energy consumption in the face milling process. The results revealed that the reduction in the energy consumption was about 20.7% when using nano fluid assisted milling [14]. Ranganathan and Senthilvelan [15] investigated the optimization of cutting parameters of stainless steel (Type 316) in hot turning using Taguchi based GRA. Pawade and Joshi [16] studied the optimization of turning parameters of Inconel 718. The Taguchi grey relational approach is used to determine the optimum process parameters that produced the minimum cutting force and surface roughness.

Researchers have worked on milling processes to improve the performances from multivarious dimensions such as the tool wear, surface quality and the cutting forces. Twardowski et al. [17] studied the effect of tool wear and tool life in the high speed milling process. They used coated carbide and cubic boron nitride cutting tools and they observed that, for cutting speeds over than 500m/min the cutting tool with edges of boron nitride must be used in the milling process. Krolczyk et al. [18] determined the surface topography of the coated carbide tool in the turning process. They found the predominant failure mechanism and the main reason behind the reduction of tool life was the flank wear of the carbide tool.

Response surface methodology, Grey relation analysis, and Taguchi methodologies were commonly adopted in the milling process to predict the surface roughness, material removal rate and machining time. The obtained results confirmed the sufficiency of these techniques to predict machinability characteristics. Grey relation analysis has been adopted to optimize cutting parameters, namely milling type, spindle speed, feed per tooth, radial depth of cut, and axial depth of cut in the high-speed milling process. Based on the results of the analysis, a proper evaluation for material removal rate and tool life has been achieved corresponding to milling type, spindle speed, and feed per tooth with 79% desirability, reported by Lu et al. [19]. Different cooling conditions assisted milling processes have been conducted to optimize quality characteristics using a response surface approach [20]. Kuram and Ozcelik [21] conducted an experimental study to find the desirable cutting parameters in micro-milling of Aluminum 7075 using a ball end tool. The optimization process for tool wear, cutting force and surface roughness has been performed using Taguchi based Grey relation analysis. The results indicated that the minimum values of tool wear, cutting force, and surface roughness were mostly affected by spindle speed, followed by feed per tooth and depth of cut. Rajeswari and Amirthagadeswaran [22] developed a model to predict the surface roughness, tool wear, cutting force and MRR using response surface methodology and grey relation analysis in end milling of aluminum composites. The experimental results revealed that the weight proportion of SiC and cutting speed are the most important parameters that influenced the machinability of material composites. Wojciechowski et al. [23] proposed a new method to improve the efficiency of the machined surfaces in the end milling process. They minimized the cutting force and increased the surface quality during the optimization process of the machining parameters.

Face milling under the semi-finishing stage was conducted in order to evaluate the surface roughness and cutting power using different lubricant conditions. Zhang and Chen [24] optimized the surface roughness in the face milling operation based on the Taguchi method. The results indicated that the depth of cut has the minimum effect on surfaces roughness compared to cutting speed and feed rate while the tool wear is statistically affected. Hashmi et al. [25] studied the single objective optimization of surface roughness using response surface methodology. The predicted model suggested that the depth of cut is the most critical parameter that affects the surface roughness in the machining process. Recently, Felhő and Kundrák [26] examined 2D and 3D roughness parameters of the machined surfaces with constant depth of cut in the face milling process. They analyzed the topography of the surface with considering the increasing of the feed per tooth and axial run-out of the inserts. The results indicated that the use of single insert face milling leads to worsen the surface with increasing the feed per tooth and the decrease in surface roughness about 1.44–7.71 times when the four-insert face milling considered. In addition to the most common machining parameters, Tseng et al. [27] predicted the effect of the cutting fluid, nose radius and cutting forces upon the surface roughness in end milling. The prediction process was included in two approaches. Firstly, the analysis of variance was implemented to determine the significant of the process parameters and the interactions of the parameters was neglected due to the impossibility of achieving them in practice. Secondly, fuzzy logic was implemented to predict the surface roughness with an accuracy of 95% compared with the experimental results. The effect of feed rate variation and insert runout errors on the surface roughness and geometric accuracy evaluated by Baek et al. [28] during the face milling process. Moreover, they developed a model for surface roughness and controlled the roughness of the machined surface by optimization of feed rate with a max material removal rate using the bisection method. The results pointed out that the relation between the surface roughness and the feed rate resulting from the runouts was nonlinear and to get the predicted roughness, the insert runout errors should be determine beforehand. In dry face milling process of selaimia et al. [29], modeling and optimization processes were achieved using a response surface methodology and desirability function. The modeling was performed with considering surface roughness, cutting power, cutting force, specific cutting force and metal removal rate. The results showed that the surface roughness is only influenced by feed per tooth while the material removal rate is influenced by both of feed per tooth and axial depth of cut. To assess the surface roughness of the surfaces sculptured by face milling, a general mathematical model was developed by Miko and Nowakowski [30] with considering tool geometry, undeformed chip thickness, tool vibrations, tool runout and tool wear. They reported that the feed rate has a significant influence on the surface roughness with small cutting tool and this significance becomes greater with a decrease in the relative displacement and deformed chip thickness as well.

From the previous studies, it is appreciable that the optimization of the tool path is still a critical key in different milling processes so that more studies should be implemented to achieve a better quality for the manufactured parts to meet the industrial requirements. The objective of this paper is to improve the surface quality in the face milling process with the use of torus end mill. This article focused on the optimization of the three most common tool path strategies taking into consideration the machining time and material removal rate. The optimization process was performed based on a grey relation technique with different process parameters; hence, the optimum tool path was found based on the conditions used in this study. The optimization procedure is explained in detail in the following sections.

## 2. Experimental Procedures

**Figure 1:**
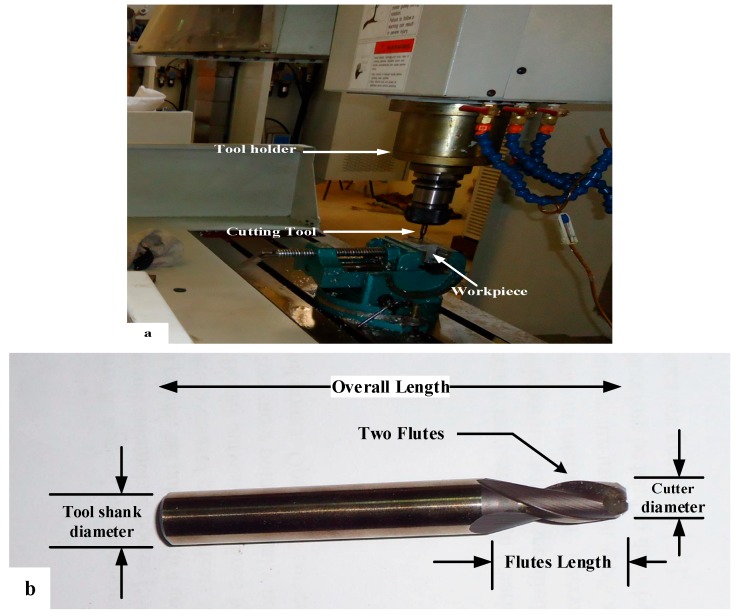
Experimental equipment: (**a**) 3-axis CNC milling machine with the workpiece and (**b**) Milling tool used for face milling.

In the present work, C-TEK CNC milling machine (Taichung, Taiwan) of model KM80D as shown in Figure 1 is used to perform the experiments of face milling with spindle speed 6000 rpm. The table of the CNC machine can move along *x*, *y*, *z* directions with a stroke length of 800 mm × 500 mm × 500 mm, respectively.

### 2.1. Material and Milling Tool

**Figure 2:**
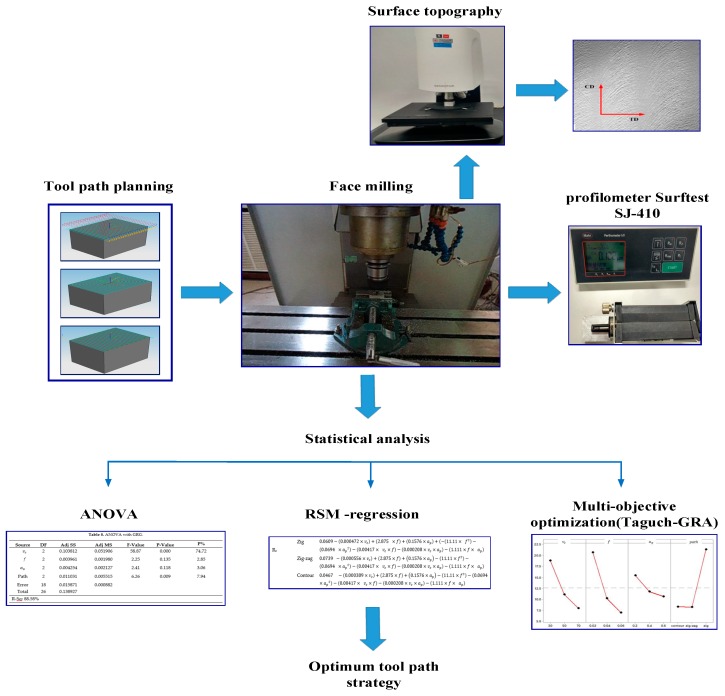
The proposed methodology.

Aluminum alloy (AL2024-T4), prepared as a rectangular block with a dimension of 40 mm × 30 mm × 30 mm, was used in a face milling operation. The chemical composition of the specimens was measured using X-MET5100 (Oxford Instruments, Abingdon, Oxfordshire, UK) and listed in Table 1. The tensile test was carried out according to ASTM B209 standard [31]. The mechanical properties are presented in Table 2. The cutting tool used in this study was Torus-end mill tool HSS (Jun-Yi, Taiwan) with 6 mm diameter, 20 mm flute length, 75 mm overall length, 10 mm shank diameter, two flutes, 2 mm corner radius, 30° helix angle and 1mm width of cut (see Figure 1b). To minimize the effect of the tool wear, new tools were used for the face milling of different sets of the tool path. The experimental set-up and the objective of the current study are shown in Figure 2.

### 2.2. Design of Experiments with Tool Path Strategies

Three quantitative input factors, i.e., cutting speed, feed per tooth, and depth of cut, and one qualitative factor, i.e., tool path strategy are adopted as input parameters, to measure the surface roughness (*R*_a_), material removal rate (MRR) and cutting time (CT) as output parameters. The selection of input factors was performed by taking into consideration the limitation of the process parameters of the machine used in this study and the values adopted in the literature. The three levels of input factors are shown in Table 3.

**Figure 3:**
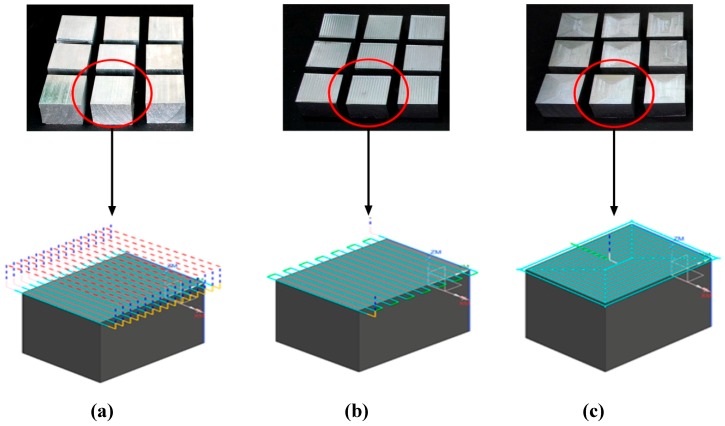
Generated tool path strategies with the machined workpiece: (**a**) Zig, (**b**) Zig-zag and (**c**) Contour.

Three strategies of tool path (i.e., zig, zig-zag, and contour) were generated using NX 10 software. The representation of these strategies with the machined workpiece is depicted in Figure 3. In order to reduce the experimentation cost, time and effort, the Taguchi method was adopted to deal with these problems. In this paper, a number of experiments (total of 27 runs) are designed based on Taguchi design which is performed in Design Expert 10. Taguchi array with the output responses is shown in Table 4.

### 2.3. Measurement of Surface Roughness

The average surface roughness parameter *R*_a_ of all the machined surfaces is measured by using a profilometer Surftest SJ-410 (Mitutoyo, Tokyo, Japan). Before starting the measurement, the tester was calibrated using the reference specimen. In the present study, 27 values of *R*_a_ were measured from workpiece surfaces at three equally divided regions, and then, the average of these values was recorded. During the roughness measurements, the tracing velocity, the sampling length, Straightness/traverse length, measuring range/resolution and cut off length were fixed at 1 mm/s, 2.5 mm, (0.3 µm/25 mm), (80 µm/0.001 µm) and 0.25 mm, respectively. It is worth mentioning that the surface roughness measurements were recorded in perpendicular to the cutting direction.

### 2.4. Material Removal Rate

The material removal rate (MRR) for the face milling operation has been calculated for each run by Equation (1) [32].

(1) $$MRR=v_{f}\times a_{e}\times a_{p}$$

where, *a*_e_ is the width of cut (mm), *a*_p_ is the depth of cut (mm), and *v*_f_ is the feed rate (mm/min).

## 3. Grey-Relation Analysis

To overcome the disadvantage of the Taguchi method which has the inability to solve a multi-response optimization problem, the Taguchi method with grey relation analysis (GRA) is combined to convert the multi-objective optimization into a single objective problem. The procedure required for the GRA analysis is delineated as follows:

After performing the experiments, the data are normalized from 0 to 1 [33]. To achieve the best surface quality with minimum time, and maximum material removal rate “the-higher-the-better” and “the-lower-the-better” conditions are chosen respectively as follows in Equations (2) and (3).

(2) $$x_{i}(k)=\frac{\max y_{i}(k)-y_{i}(k)}{\max y_{i}(k)-\min y_{i}(k)}$$

(3) $$x_{i}(k)=\frac{y_{i}(k)-\min y_{i}(k)}{\max y_{i}(k)-\min y_{i}(k)}$$

where *x_i_*(*k*) is the response after linear normalization, *y_i_*(*k*) is the experimental response, min *y_i_*(*k*) is the smallest value of *y_i_*(*k*) and max *y_i_*(*k*) is the largest value of *y_i_*(*k*).

The following step is used for calculating the grey relation coefficient, which represents the relevance between the ideal and actual experimental results after the normalization process; the Equation (4) can be used

(4) $$\xi_{i}(k)=\frac{\Delta_{\min}+\phi \Delta_{\max}}{\Delta_{0i}(k)+\phi \Delta_{\max}}$$

where Δ_0*i*_(*k*) represents the absolute value of the deviation between *y*_0_(*k*),and *y_i_*(*k*), Δ_min_ and Δ_max_ are the smallest and largest values of Δ_0*i*_(*k*) respectively. ϕ is the distinguishing coefficient. The value of ϕ is defined as ϕ ∈ [0,1].The different values of the ϕ give different values of grey relation coefficients; however, the rank order of GRC, is, always the same [34]. In this study, the value of ϕ is considered as 0.5.

The last step is calculating the average value of GRC (i.e., GRG (Υ*_i_*)) which is defined as in Equation (5).

(5) $$\gamma_{i}=\sum_{k=1}^{n}w_{k}\xi_{i}(k)$$

where *w_k_* indicates the weight of the *k*th experimental response. In the current study, the weights for surface roughness, material removal rate, and cutting time are 0.3626, 0.2928, and 0.3446 respectively which are calculated using the entropy method [35], as shown in Table 5. The max value of GRG indicates that the process parameters at that value are close to the optimum one [36]. In Table 5, the max value of GRG is corresponding to trail No. 9, which refers to A3B3C3D1 as the best combination of input factors.

## 4. RSM and Optimization

### 4.1. Response Surface Regression

Response surface methodology (RSM) represents a good approach that can be used to determine the optimum performance of the experimental response that is influenced by several factors. In this study, a second order polynomial equation is used and represented by Equation (6) [37].

(6) $$y=b_{0}+\sum_{i=1}^{k}b_{i}X_{i}+\sum_{i=1}^{k}b_{ii}X_{i}^{2}+\sum_{i≺j}^{k}b_{ij}X_{i}X_{j}$$

where *y* is *r*th response (in the current study: *R*_a_, MRR and CT), *X* is input factor (in the current study: *v*_c_, *f* and *a*_p_) and *b* regression coefficient, respectively. On the other hand, the regression coefficients have been estimated by MINITAB 18 software.

The type of the tool path is a qualitative factor, and it cannot be treated as quantifying factor. Consequently, a regression model must be made for each strategy of the tool path. A regression model for surface roughness, material removal rate and cutting time regarding tool path strategies are depicted as equations in Table 6.

### 4.2. Evaluation of Optimum Experimental Run

The average value of GRG has been computed with respect to the different levels of machining parameters as shown in Table 7. The maximum value of GRG indicates the best performance. From Table 7, the largest value of GRG at level 3 for cutting speed followed by tool path, whereas the minimum value for depth of cut at the same level. The difference between the max and min of each factor at different levels has been calculated. The max difference showed that the cutting speed has the most significant influence on GRG while the feed rate has the least influence.

### 4.3. Contribution of Milling Parameters

To determine the contribution for each input factors on the output performance, the analysis of variance (ANOVA) has been conducted on the grey relational grade to achieve this goal. Table 8 lists the results obtained from this analysis.

The ANOVA results refer to the most significant factor (i.e., v_c) with contribution 74.72% followed by tool path strategy, whereas the feed per tooth has the least effect on the multi-objective response (see Table 8). This finding is in agreement with reference [8], in which the significance of feed is about 0.95 which considered the smallest effect in the analysis.

### 4.4. Experimental Verification of Optimum Levels of Input Parameters

It is necessary to confirm and evaluate the performance of the predicted models with respect to the optimum machining parameters, which are calculated based on GRG analysis. Therefore, the predicted GRG was computed first by using the following expression [38].

(7) $$G_{p}=G_{m}+\sum_{i=1}^{k}\left(G_{i}-G_{m}\right)$$

where *G*_p_, *G*_m_, and *G_i_* are the predicted value of GRG, the total average value of GRG, and the mean value of GRG at best level of input factors.

After that, the experimental test has been conducted at the levels of predicted GRG. Table 9 presents the experimental confirmation results of optimum process factors.

According to the Equation (7), the predicted value of GRG has been calculated as shown in Table 9, and the estimated value of GRG from the experimental confirmation test was 0.7824, which indicates an improvement about 7.162% from the predicted one.

## 5. Discussion

From the past section, the prediction and experimental observations confirmed that the third level of input parameters provided the optimum performance, which confirmed the adequacy of this study. However, these findings should be addressed in detail to provide a better understanding of the milling process and characteristic performance. In this section, the analysis of responses (i.e., *R*_a_, MRR, and CT) has been studied based on ANOVA method by considering the interaction and mean effect plots, which provide more information about the interaction of different process parameters on the response in addition to the effect of each input factor separately.

### 5.1. Surface Topography for Face Milling Process

**Figure 4:**
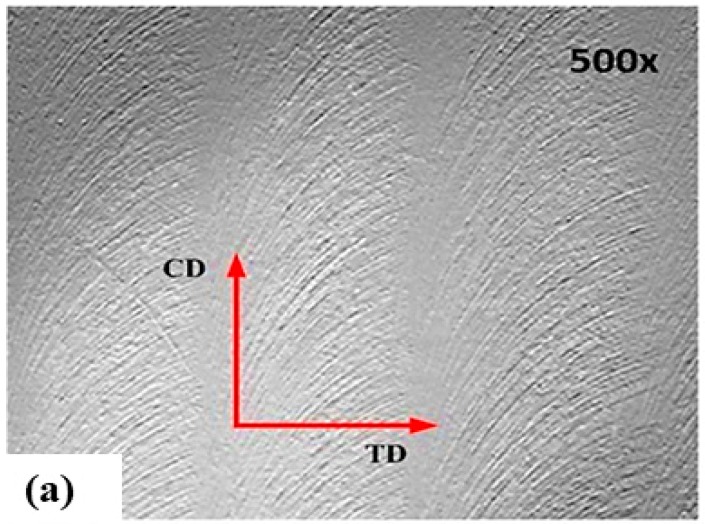

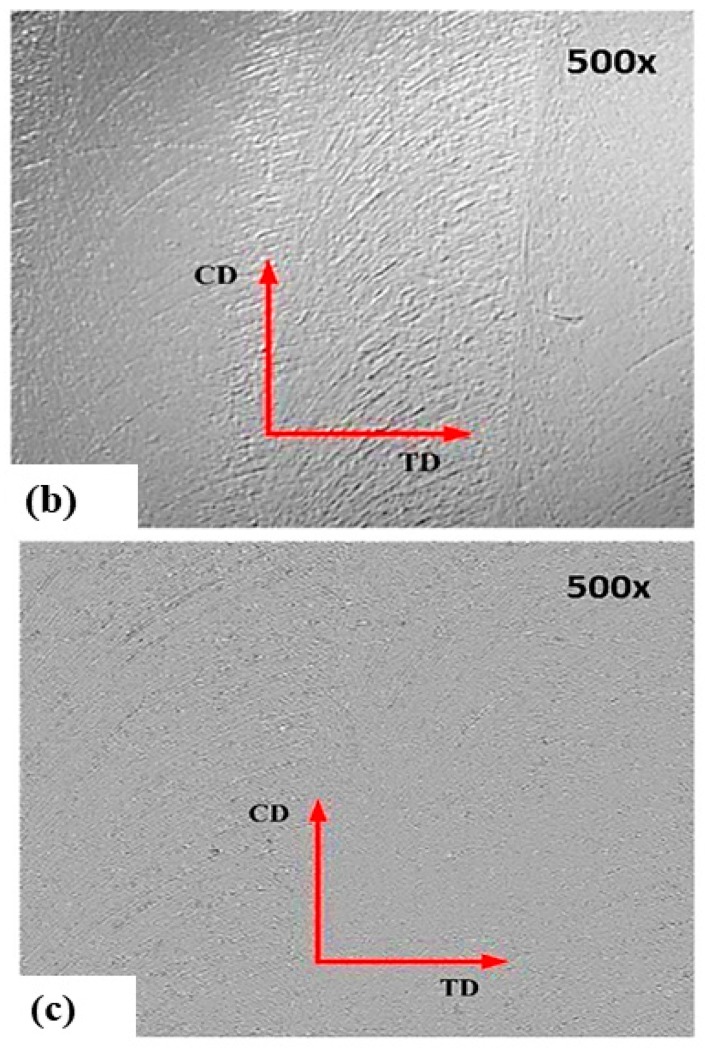
Surface topography for different tool path strategies: (**a**) zig, (**b**) zig-zag and (**c**) contour.

The obtained surface topography is shown in Figure 4a–c for zig, zig-zag, and contour tool path strategies, respectively. Here, CD refers to the cutting direction while TD refers to the transverse cutting direction. Surface topography was conducted using non-contact 3D surface profilometer (SNEOX) with magnification ×1000 for face milling process of Al2024. In Figure 4a–c the topography under different conditions for different cutting path strategies was performed with SENSOFAR. Regarding the zig and zig-zag topography (Figure 4a,b), the one generated by the contour strategy (Figure 4c) shows some clear differences. In the contour strategy, the cutting direction does not clearly index. The peaks are not obvious compared with the surfaces generated under other cutting strategies. Moreover, the pattern of the contour path is more uniform, and the reasons behind these observations were studied extensively by reference [6].

### 5.2. Analysis of Surface Roughness

**Figure 5:**
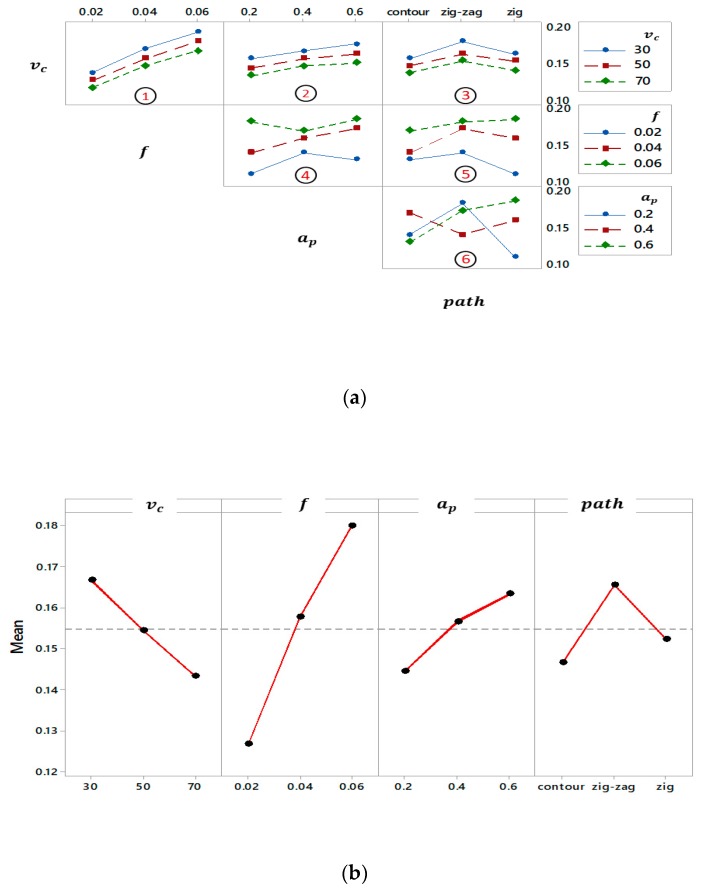
Graphical analysis of *R*_a_: (**a**) Interaction plot and (**b**) Main effects plot.

To study the influence of different factors with each other on the surface roughness, the interaction plot which is divided into subplots has been implemented as shown in Figure 5a. The interaction effects of process parameters are obvious except subplots 1,2, and 3.

The effect of feed per tooth, depth of cut and tool path (TP) are the same for cutting speed of 30, 50 and 70 m/min (see Figure 5a, subplot 3), however, the effect of other factors stay invariant. It means face milling of zig-zag tool path for three levels of cutting speed results in higher surface roughness. The surface degradation in the case of the zig-zag tool path refers to keeping the chip formation between the milling tool and the machined surface, which leads to an increase the surface roughness [8].

Both the cutting speed and feed per tooth have a similar effect when other parameters are kept constant. It means that different levels of cutting speed have the same behavior even the level of feed per tooth is changed. Moreover, the surface roughness increases with an increasing feed per tooth for three levels of cutting speed. In addition, the same behavior is observed for depth of the cut and cutting speed. Therefore, parameter combinations that can alter the surface roughness with identical tendency are *f*-*v*_c_, *a*_p_-*v*_c_, and TP-*v*_c_, while other parameter series have mutual influences and act inversely to each other when other factors are considered unchanged.

The main influence of input parameters on surface roughness is illustrated in Figure 5b; it is observed that the surface roughness increases about 0.04 µm when the feed per tooth and depth of cut increase, while a cutting speed of 70 m/min provides lower surface roughness compared to 30 m/min.

The same effect has been noticed in the work of reference [8] for Al6005A for 6-axis robotic machining regarding the cutting speed and feed; however, the relationship between surface roughness and cutting speed has inversely trended as reported by reference [39]. In micro milling [40] of composite materials, the increment of cutting speed decreased the surface roughness, and this result is in agreement with Figure 5a. The reason behind the improvement of surface roughness with increasing cutting speed is that the increase of the heat between the cutting tool and the machined surface softened the machining area. In addition, the reduction of built-up-edge (BUE) with increasing cutting speed also contributed [41,42]. In this study, the surface roughness decreases with an increasing cutting speed and a decreasing feed per tooth and depth of cut. In fact, the increase of feed per tooth led to increase the vibration of the tool hence reduced the quality of the machined surfaces [43]. Similar behavior is observed in the work of reference [21] for micro-milling of Al7075. Furthermore, the implementation of the face milling process with the contour tool path causes the surface roughness to decrease. Hence, it can be concluded that a higher level of feed per tooth and depth of cut and also a lower level of cutting speed with a medium level of tool path results in a higher surface roughness.

### 5.3. Analysis of Material Removal Rate

**Figure 6:**
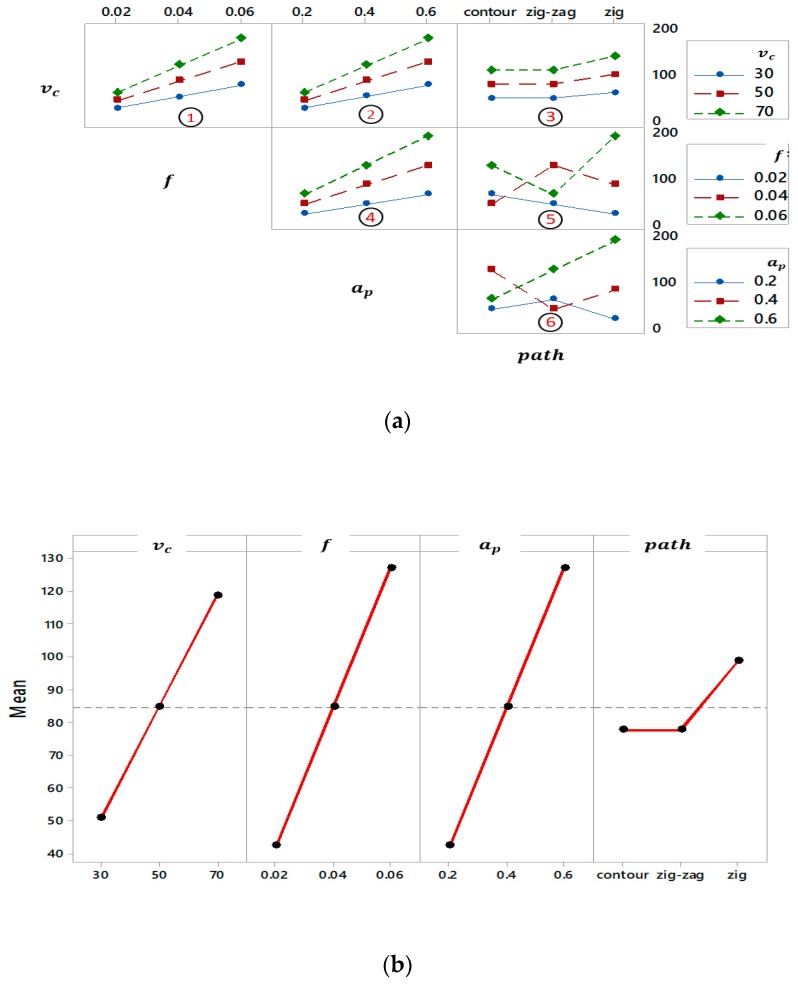
Productivity (MRR) analysis of milling: (**a**) Interaction plot and (**b**) Main effects plot.

In this section, an interaction plot for the material removal rate is presented in Figure 6a. The effects of the tool path (TP) strategy (zig-zag and contour) are the same for cutting speed of 30, 50, and 70 m/min. Also, the effect of both tool paths is not modified by the cutting speed (see Figure 6a, subplot 3). It means that the same trend for TP-*v*_c_ while the other factors are kept constant and the behavior of contour and zig-zag tool path do not affect the MRR even by changing the cutting speed. On the other hand, the subplots 5 and 6 (i.e., TP-*f* and TP-*a*_p_) have interactions and, they affect each other inversely. For example, the tool path and feed per tooth have interactions while other factors are unchanged. In contrast, the subplots (1, 2, 3 and 4) have the same trend regarding MRR when the other parameters kept constant. Figure 6b demonstrates the main effect of every factor on the material removal rate after face the milling process. When the cutting speed, feed per tooth, and depth of cut increase, MRR increases as well. However, for the medium and highest levels of tool path (i.e., zig, zig-zag, and contour), the MRR is still unchanged. Similar ascendant impact of cutting speed, feed per tooth, and depth of cut on MRR are observed in reference [15]. As a result, the maximum value of MRR can be acquired when the input parameters (i.e., *v*_c_, *f*, *a*_p_ and TP) reach their highest level.

### 5.4. Analysis of Machining Time

**Figure 7:**
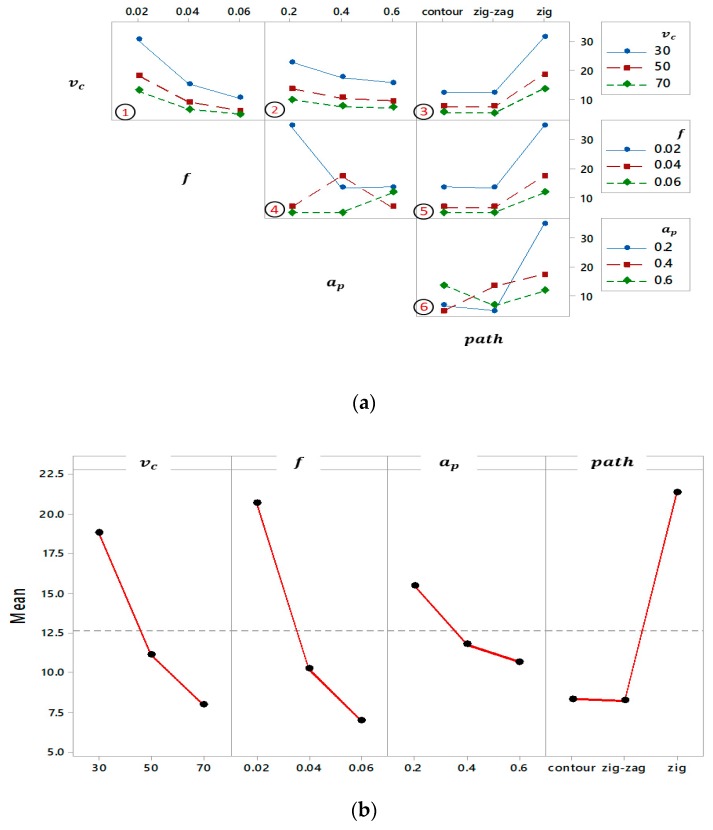
Time analysis for milling process: (**a**) Interaction plot and (**b**) Main effects plot.

The interaction effects of different levels of machining parameters on the machining time are shown in Figure 7a. The subplots (4 and 6) have different interactions; however, the other parameter sets (see Figure 7a, subplot 1, 2, 3, and 5) have the same behavior when the other factors are considered unchanged. For instance, the effect of both medium and high level of the path is the same for three levels of cutting speed and feed per tooth. In addition, the effect of the aforementioned path is not altered by cutting speed and feed per tooth (see Figure 7a, subplot 3 and 5). As a result of TP- *v*_c_ and TP-*f* have an identical effect when the other factors are kept constant; hence, the changing path type (i.e., zig-zag and contour) does not affect the machining time regardless of the changes of *v*_c_ and *a*_p_.

Figure 7b presents the main effect of process parameters on the milling time, the lowest level of each factor (i.e., cutting speed, feed per tooth, and depth of cut) produce the minimum value of milling time except the tool path, for the zig path the time increased rapidly while it is kept constant for the zig-zag and contour paths [44].

### 5.5. Grey Relational Grade

**Figure 8:**
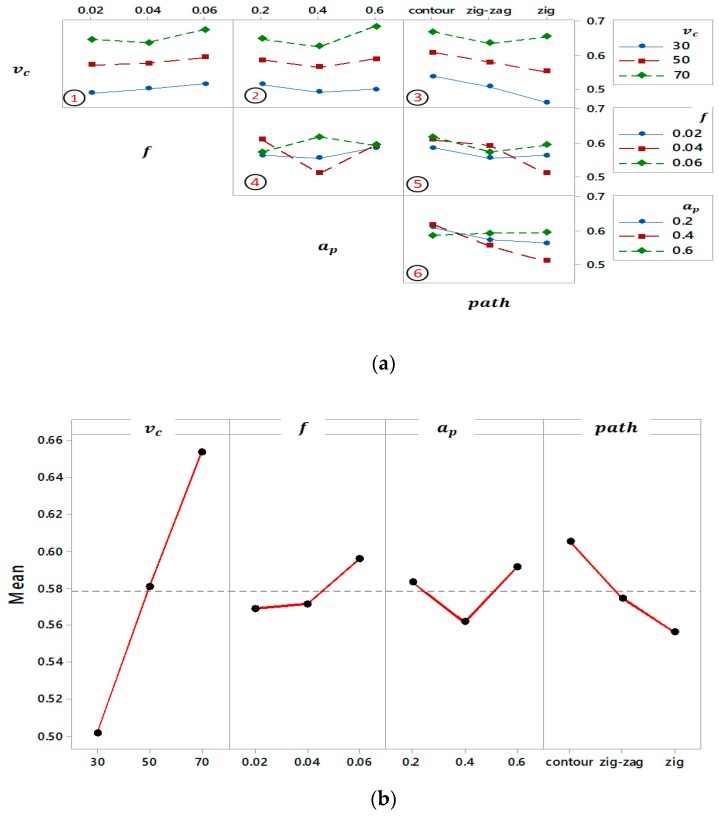
Grey relational grade: (**a**) Interaction plot and (**b**) Main effects plot.

To evaluate the interaction of process parameters in detail, the GRG analysis was used as shown in Figure 8a. The subplots 1,2 and 3 had the same trend when the other factors considered constant. Moreover, there is clear interaction in subplots 4, 5 and 6 which affect each other in different ways. From subplot 3 it can be seen that the effect of second and third levels of tool path is approximately the same under three levels of cutting speed, hence the effect of these two levels of tool path doesn’t change with cutting speed, while in the subplot 5 the trend of different levels of tool path is the same under the first and third levels of feed per tooth. Consequently, the medium and maximum levels of the tool path in TP-*v*_c_ and TP-*f* have a similar effect when the other factors are kept unchanged; hence, the changing path type (i.e., zig-zag and contour) does not affect the GRG regardless of the changes of *v*_c_ and *a*_p_.

Because the GRA can only evaluate the optimum combination from the designed plan, the average grey relational analysis is used to find the significant level of each input factor while considering the different levels of the process parameters in the current study. From Table 7 and Figure 8b, the effect of three levels of milling parameters on GRG was estimated. The highest value of GRG refers to the optimum level of process parameters as portrayed in Figure 8b. The optimum setting of process parameters for maximizing the multi-response characteristics was a cutting speed of 70 m/min (level 3), feed per tooth of 0.06 mm/tooth (level 3), depth of cut of 0.6 mm (level 3) and contour strategy (level 3) for tool path.

From the above analysis, it can be concluded that the contour tool path can be adopted in the face milling parts in synchronism with the observations of reference [44], which confirmed the ability of this strategy not only to improve the surface roughness but also to reduce the energy demand and machining time.

## 6. Conclusions

In this study, multi-objective optimization has been conducted for face milling process of Al-2024. The influences of different cutting parameters are investigated which include the cutting speed, feed per tooth, depth of cut, and tool path strategy. A series of experiments have been carried out to achieve a good surface quality in less machining time. The summary of the current study is drawn as follows:

From the ANOVA analysis, the cutting speed has been found as the main influencing factor on the response with 74.72% contribution. Following cutting speed, the tool path strategy exerted 7.94% contribution, and then the depth-of-cut exhibited 3.06% contribution, and lastly, the feed per tooth has 2.85% influences.

- The best machining performance was obtained at a cutting speed of 70 m/min, feed per tooth of 0.06 mm, and depth of cut of 0.6 mm when the cutting was conducted in a contour tool path strategy. The optimum combination was validated with the confirmation test that has ensured the optimum performance of the responses.
- The milling time for the zig tool-path strategy rapidly increased for the machined parts compared with the other tool-path strategies. On the other hand, the contour tool path strategy can be considered as the best tool path strategy that provided the minimum surface roughness and cutting time with more uniform surface topography.
- The contributions of the current study and prediction models can be considered as a guideline in the face milling process, especially when good quality and a minimum milling time are required.
- It is worth mentioning that the results of this study are proper under the cutting conditions used; any change in the machining tool or process conditions may affect the output performance.
- The current work is applicable in the metal processing industry to get good quality parts with the least time, which will save energy and cost. For future work, the proposed work strategy will be applied to free form surfaces of difficult to cut materials and best tool path will be determined.

## Figures and Tables

**Table 1:** The chemical composition of Al2024-T4 alloy.

Elements	Si	Fe	Cu	Mn	Mg	Pb	Zn	Ni	Al
Composition (wt.%)	0.34	0.35	5.03	0.83	0.75	0.07	0.14	0.01	92.48

**Table 2:** Mechanical properties of Al2024-T4 alloy.

Tensile Strength (MPa)	Yield Strength (MPa)	Elongation (%)	Modulus of Elasticity (GPa)
469	324	20%	73.1

**Table 3:** Factors and levels of the milling process.

Quantify Input Factors and Qualify		Levels		
		1	2	3
Cutting speed (m/min)	*v*_c_	30	50	70
Feed per tooth (mm/tooth)	*f*	0.02	0.04	0.06
Depth of cut (mm)	*a*_p_	0.2	0.4	0.6
Tool path strategy	TP	Zig	Zig-zag	Contour

**Table 4:** Experimental results.

Sl. No	Orthogonal Array				Measured Performance		
	*v*_c_ (m/min)	*f* (mm/tooth)	*a*_p_ (mm)	TP	*R*_a_ (µm)	MRR(mm^3^/min)	CT (min)
1	30	0.02	0.2	zig	0.12	12.8	52
2	30	0.04	0.4	zig	0.17	50.8	26
3	30	0.06	0.6	zig	0.2	114.6	17
4	50	0.02	0.2	zig	0.11	21.2	31
5	50	0.04	0.4	zig	0.16	84.8	15
6	50	0.06	0.6	zig	0.19	190.8	10
7	70	0.02	0.2	zig	0.1	29.6	22
8	70	0.04	0.4	zig	0.15	118.8	11
9	70	0.06	0.6	zig	0.17	267	8
10	30	0.02	0.4	zig-zag	0.15	25.6	20
11	30	0.04	0.6	zig-zag	0.19	76.2	10
12	30	0.06	0.2	zig-zag	0.2	38.2	7
13	50	0.02	0.4	zig-zag	0.14	42.4	12
14	50	0.04	0.6	zig-zag	0.17	127.2	6
15	50	0.06	0.2	zig-zag	0.18	63.6	4
16	70	0.02	0.4	zig-zag	0.13	59.2	8
17	70	0.04	0.6	zig-zag	0.16	178.2	4
18	70	0.06	0.2	zig-zag	0.17	89	3
19	30	0.02	0.6	contour	0.14	38.4	20
20	30	0.04	0.2	contour	0.15	25.4	10
21	30	0.06	0.4	contour	0.18	76.4	7
22	50	0.02	0.6	contour	0.13	63.6	12
23	50	0.04	0.2	contour	0.14	42.4	6
24	50	0.06	0.4	contour	0.17	127.2	4
25	70	0.02	0.6	contour	0.12	88.8	9
26	70	0.04	0.2	contour	0.13	59.4	4
27	70	0.06	0.4	contour	0.16	178	3

**Table 5:** Calculated grey relational coefficient with different weights and GRG.

Run No.	GRC			GRG	Rank
	*R*_a_	MRR	CT		
**1**	0.7143	0.3333	0.3333	0.4715	26
2	0.4167	0.3702	0.5158	0.4372	27
3	0.3333	0.4547	0.6364	0.4733	25
4	0.8333	0.3408	0.4667	0.5628	16
5	0.4545	0.4109	0.6712	0.5164	21
6	0.3571	0.6252	0.7778	0.5806	14
7	1.0000	0.3487	0.5632	0.6588	5
8	0.5000	0.4617	0.7538	0.5762	15
**9**	**0.4167**	**1.0000**	**0.8305**	**0.7301**	**1**
10	0.5000	0.3449	0.5904	0.4857	24
11	0.3571	0.3998	0.7778	0.5146	22
12	0.3333	0.3571	0.8596	0.5216	20
13	0.5556	0.3614	0.7313	0.5593	17
14	0.4167	0.4762	0.8909	0.5975	11
15	0.3846	0.3846	0.9608	0.5831	13
16	0.6250	0.3795	0.8305	0.6239	7
17	0.4545	0.5887	0.9608	0.6683	4
18	0.4167	0.4166	1.0000	0.6176	9
19	0.5556	0.3573	0.5904	0.5095	23
20	0.5000	0.3447	0.7778	0.5502	19
21	0.3846	0.4001	0.8596	0.5528	18
22	0.6250	0.3846	0.7313	0.5912	12
23	0.5556	0.3614	0.8909	0.6142	10
24	0.4167	0.4762	0.9608	0.6216	8
25	0.7143	0.4163	0.8033	0.6577	6
26	0.6250	0.3797	0.9608	0.6689	3
27	0.4545	0.5882	1.0000	0.6816	2

**Table 6:** Regression models for experimental response.

Response	Path	Model
*R*_a_	Zig	$0.0609-\left(0.000472\times v_{c}\right)+\left(2.875\times f\right)+\left(0.1576\times a_{p}\right)+(-\left(11.11\times f^{2}\right)-$ $\left(0.0694\times {a_{p}}^{2}\right)-\left(0.00417\times v_{c}\times f\right)-\left(0.000208\times v_{c}\times a_{p}\right)-\left(1.111\times f\times a_{p}\right)$
	Zig-zag	$0.0739-\left(0.000556\times v_{c}\right)+\left(2.875\times f\right)+\left(0.1576\times a_{p}\right)-\left(11.11\times f^{2}\right)-$ $\left(0.0694\times {a_{p}}^{2}\right)-\left(0.00417\times v_{c}\times f\right)-\left(0.000208\times v_{c}\times a_{p}\right)-\left(1.111\times f\times a_{p}\right)$
	Contour	$0.0467-\left(0.000389\times v_{c}\right)+\left(2.875\times f\right)+\left(0.1576\times a_{p}\right)-\left(11.11\times f^{2}\right)-(0.0694$ $\times {a_{p}}^{2})-\left(0.00417\times v_{c}\times f\right)-\left(0.000208\times v_{c}\times a_{p}\right)-\left(1.111\times f\times a_{p}\right)$
MRR	Zig	$70.8-\left(1.417\times v_{c}\right)-\left(2125\times f\right)-\left(211.7\times a_{p}\right)+\left(42.50\times v_{c}\times f\right)+$ $\left(4.233\times v_{c}\times a_{p}\right)+\left(5300\times f\times a_{p}\right)$
	Zig-zag	$92-\left(1.840\times v_{c}\right)-\left(2125\times f\right)-\left(211.7\times a_{p}\right)+\left(42.50\times v_{c}\times f\right)+$ $\left(4.233\times v_{c}\times a_{p}\right)+\left(5300\times f\times a_{p}\right)$
	Contour	$92.2-\left(1.840\times v_{c}\right)-\left(2125\times f\right)-\left(211.7\times a_{p}\right)+\left(42.50\times v_{c}\times f\right)+$ $\left(4.233\times v_{c}\times a_{p}\right)+\left(5300\times f\times a_{p}\right)$
CT	Zig	$115.8-\left(1.436\times v_{c}\right)-\left(1550\times f\right)-\left(63.2\times a_{p}\right)+\left(0.00569\times {v_{c}}^{2}\right)+\left(9028\times f^{2}\right)$ $+\left(31.9\times {a_{p}}^{2}\right)+\left(7.50\times v_{c}\times f\right)+\left(0.292\times v_{c}\times a_{p}\right)+\left(278\times f\times a_{p}\right)$
	Zig-zag	$90.5-\left(1.169\times v_{c}\right)-\left(1550\times f\right)-\left(63.2\times a_{p}\right)+\left(0.00569\times {v_{c}}^{2}\right)+\left(9028\times f^{2}\right)$ $+\left(31.9\times {a_{p}}^{2}\right)+\left(7.50\times v_{c}\times f\right)+\left(0.292\times v_{c}\times a_{p}\right)+\left(278\times f\times a_{p}\right)$
	Contour	$90.2-\left(1.161\times v_{c}\right)-\left(1550\times f\right)-\left(63.2\times a_{p}\right)+\left(0.00569\times {v_{c}}^{2}\right)+$ $\left(9028\times f^{2}\right)+\left(31.9\times {a_{p}}^{2}\right)+\left(7.50\times v_{c}\times f\right)+\left(0.292\times v_{c}\times a_{p}\right)+\left(278\times f\times a_{p}\right)$

**Table 7:** Average values of GRG at different levels of machining parameters.

Identification	Cutting Speed	Feed Rate	Depth of Cut	Tool Path
Level 1	0.50183	0.56893	0.5832	0.55631
Level 2	0.58074	0.57151	0.56164	0.57462
Level 3	0.65367	0.59581	0.59141	0.60531
Difference	0.15184	0.02688	0.02976	0.04899
Rank	1	4	3	2
Optimised factor	70	0.06	0.6	Contour

**Table 8:** ANOVA with GRG.

Source	DF	Adj SS	Adj MS	*F*-Value	*p*-Value	P%
$v_{c}$	2	0.103812	0.051906	58.87	0.000	74.72
*f*	2	0.003961	0.001980	2.25	0.135	2.85
$a_{p}$	2	0.004254	0.002127	2.41	0.118	3.06
Path	2	0.011031	0.005515	6.26	0.009	7.94
Error	18	0.015871	0.000882	–	–	–
Total	26	0.138927	–	–	–	–
*R*-*S*_q_: 88.58%						

**Table 9:** Confirmation results for the response.

Initial Cutting Conditions	Optimal Milling Conditions		
	Predicted Results		Experimental Results
Levels	A3B3C3D1	A3B3C3D3	A3B3C3D3
Surface roughness (µm)	0.17	–	0.14
MRR(mm^3^/min)	267	–	267
Milling time, CT(min)	8	–	7.8
GRG	0.7301	0.7099	0.7824
The % improvement in GRG = 7.162			
